# State Registration of Trained Nurses

**Published:** 1889-11

**Authors:** 


### State Registration of Trained Nurses.

A meeting recently convened in London for the purpose of considering what is asserted by the British Nurses' Association to be a question of national importance—namely, the State registration of trained nurses. The objects of the association are to obtain a royal charter for the legal registration of qualified nurses in the same form as the Medical Register. It was urged that the granting of such charter would protect the public from unskilled workers, who by their ignorance cause much suffering and even danger to the sick.

Ice in the Sick-room.—The *Sanitarian* says: "A saucerful of shaved ice may be preserved for twenty-four hours with the thermometer in the room at 90° F., if the following precautions are observed: Put the saucer containing the ice in a soup plate and cover it with another. Place the soup plates thus arranged on a good heavy pillow and cover it with another pillow, pressing the pillows so that the plates are completely imbedded in them. An old jack plane set deep is a most excellent thing with which to shave ice. It should be turned bottom upward and the ice shoved backward and forward over the cutter."

